# Neuroendocrine control of appetite and metabolism

**DOI:** 10.1038/s12276-021-00597-9

**Published:** 2021-04-09

**Authors:** Eun-Seon Yoo, Jieun Yu, Jong-Woo Sohn

**Affiliations:** Department of Biological Sciences, Korea Advanced Institute of Science and Technology, Daejeon, South Korea

**Keywords:** Hypothalamus, Homeostasis

## Abstract

Body homeostasis is predominantly controlled by hormones secreted by endocrine organs. The central nervous system contains several important endocrine structures, including the hypothalamic-pituitary axis. Conventionally, neurohormones released by the hypothalamus and the pituitary gland (hypophysis) have received much attention owing to the unique functions of the end hormones released by their target peripheral organs (e.g., glucocorticoids released by the adrenal glands). Recent advances in mouse genetics have revealed several important metabolic functions of hypothalamic neurohormone-expressing cells, many of which are not readily explained by the action of the corresponding classical downstream hormones. Notably, the newly identified functions are better explained by the action of conventional neurotransmitters (e.g., glutamate and GABA) that constitute a neuronal circuit. In this review, we discuss the regulation of appetite and metabolism by hypothalamic neurohormone-expressing cells, with a focus on the distinct contributions of neurohormones and neurotransmitters released by these neurons.

## Metabolism: Dual function for neurohormone-producing cells in the brain

Signaling molecules produced by the brain’s hypothalamus function both as neurotransmitters (within the central nervous system) and hormones (throughout the rest of the body) to regulate appetite and metabolism. Jong-Woo Sohn and colleagues from the Korea Advanced Institute of Science and Technology in Daejeon, South Korea, summarize the well-established ways in which certain hypothalamic cells interact with parts of the pituitary gland in the brain to control the activity of hormones involved in feeding behaviors and energy balances.The same cells can also impact appetite and metabolism in non-hormonal ways. New research has shown that neurohormone-producing cells in the hypothalamus can form connections with appetite-associated neurons and communicate via neurotransmitters. A deeper understanding of this process could lead to new therapies for obesity, diabetes and other metabolic disorders.

## Introduction

The hypothalamus is a vital region of the brain that regulates whole-body homeostasis. Hypothalamic neuroendocrine cells control homeostasis through the production and secretion of neurohormones into the general circulation^1^. Similar to other endocrine organs, some hypothalamic neuroendocrine cells secrete end hormones (e.g., oxytocin [OXT] and vasopressin [VP]), which are delivered to their target organs, where they produce specific effects. Other types of hypothalamic neuroendocrine cells secrete releasing hormones, such as corticotropin-releasing hormone (CRH). Releasing hormones are secreted into the hypophyseal portal system and consequently excite a second population of neuroendocrine cells in the anterior pituitary gland (adenohypophysis) to secrete stimulating hormones, such as adrenocorticotropic hormone (ACTH). This type of functional connection between hypothalamic and pituitary neuroendocrine cells is referred to as the hypothalamic-pituitary (HP) axis, which constitutes a major component of the hypothalamic neuroendocrine system.

The HP axis provides important humoral responses to challenges such as stress and cold. The HP-adrenal (HPA) axis releases glucocorticoids from the adrenal cortex in response to stress, and the HP-thyroid (HPT) axis releases thyroid hormones from the thyroid gland in response to cold^2,3^. Hormones released by the HPA and HPT axes were also shown to regulate appetite and metabolism. Glucocorticoids were shown to increase appetite and affect glucose homeostasis^4^, and thyroid hormones tend to promote appetite, increase heat generation, and reduce body weight^5^. Human diseases associated with dysfunction of the HPA axis (e.g., Cushing syndrome) and the HPT axis (e.g., Graves’ disease) are characterized by abnormal feeding behavior and/or perturbed metabolism, which confirms the above-mentioned effects of these hormones^6,7^. Additionally, CRH and thyrotropin-releasing hormone (TRH) are reported to control appetite and metabolism^8–10^. These findings highlight the potential importance of hypothalamic CRH and TRH neurons as well as the HPA and HPT axes in the regulation of energy homeostasis and metabolism.

All neurons that express neurohormones are not “neuroendocrine” cells; some neurohormone-expressing neurons (e.g., CRH and TRH neurons) do not project to the median eminence^11,12^. Therefore, neurohormone-expressing cells comprise neuroendocrine and non-neuroendocrine cells, and the term “neuroendocrine cells” may only be used to refer to neurons that project to the median eminence and release neurohormones. However, a subset of CRH neurons located in the paraventricular nucleus of the hypothalamus (PVH) labeled with peripherally injected fluorogold also showed retrograde labeling of fluorescent beads injected into the lateral hypothalamic area (LHA)^13^. These results suggest that neuroendocrine and non-neuroendocrine cells are not always completely segregated within a single population of neurohormone-expressing cells. However, throughout this review, we use the expression “neuroendocrine cells” only to refer to neuroendocrine neurohormone-expressing cells.

The non-neuroendocrine neurohormone-expressing cells of the hypothalamus constitute a neural circuitry that utilizes conventional neurotransmitters. For example, previous studies have reported that TRH neurons within the PVH form glutamatergic synapses on orexigenic (appetite-promoting) agouti-related peptide (AgRP) neurons within the arcuate nucleus of the hypothalamus (ARH), the stimulation of which increases appetite^14^. Moreover, PVH CRH neurons send glutamatergic fibers to neurons of the LHA, which is reportedly involved in stress behaviors^13^. OXT neurons within the PVH were also shown to transmit glutamatergic projections to the lateral parabrachial nucleus (PBN) to regulate fluid intake^15^. Therefore, it is important to consider the neural (non-neuroendocrine) circuitry collectively with the neuroendocrine axis to gain a deeper and complete understanding of the role of hypothalamic neurohormone-expressing cells.

In this review, we summarize the endocrine function of selected hypothalamic neurohormone-expressing cells and the relevant HP axis that regulates appetite and metabolism. We also discuss recent studies that investigated the role of conventional neurotransmitters released by these neurons.

## Hypothalamic neuroendocrine cells and metabolic function

**Fig. 1:**
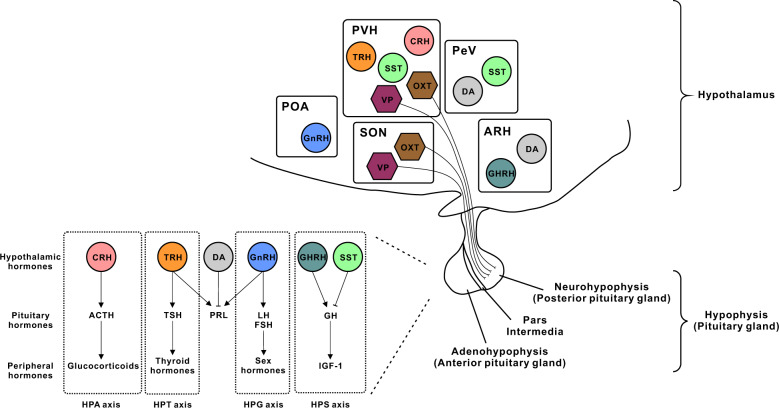
Hypothalamic neuroendocrine cells and hormones. Hypothalamic neuroendocrine cells regulate the adenohypophysis via the HP axis and the neurohypophysis through their axons. Note that the axons of the OXT and VP neurons constitute the neurohypophysis. The axons of other hypothalamic neurons are not shown for clarity. See the text for abbreviations.

Hypothalamic neuroendocrine cells release neurohormones that regulate the homeostatic function of the pituitary gland (hypophysis) (Fig. 1). Usually, hypothalamic neuroendocrine cells are classified depending on the mode of control of pituitary hormone secretion. A specific class of hypothalamic neuroendocrine cells synthesizes and transports hormones to axon terminals, which constitute the posterior pituitary gland (neurohypophysis). These posterior pituitary hormones include OXT and VP (also called arginine VP [AVP] or anti-diuretic hormone [ADH]) (Fig. 1). Neuroendocrine cells that release OXT and VP are located in both the PVH and the supraoptic nucleus (SON)^16^. OXT causes contraction of the smooth muscles of the uterus and mammary gland, and VP acts on the kidneys to promote water reabsorption^17^. These posterior pituitary hormones and the OXT and VP neurons are also known to control appetite and metabolism. For example, OXT administration causes anorexia^18^, and hypothalamic lesions of OXT neurons result in hyperphagia and obesity^19^. OXT neurons within the PVH were shown to be inhibited by AgRP neurons within the ARH^20^. OXT also produces several beneficial metabolic effects, such as an increase in energy expenditure and promotion of lipolysis^18^. On the other hand, experimental evidence suggests that VP suppresses food intake^21^. Recent studies in mouse models have reported that chemogenetic activation of VP neurons within the PVH induced anorexia, whereas chemogenetic inhibition of these neurons partially reversed anorexia induced by MTII, a melanocortin-4 receptor (MC4R) agonist^22^. Reportedly, chemogenetic activation of VP neurons located in the PVH, SON, and suprachiasmatic nucleus reduces food intake in rats^23^, further supporting the anorexigenic (appetite-suppressing) property of VP neurons.

Another class of hypothalamic neuroendocrine cells regulates the secretion of hormones from the adenohypophysis via the hypophyseal portal system. These anterior pituitary hormones include CRH, TRH, growth hormone-releasing hormone (GHRH), somatostatin (SST), gonadotropin-releasing hormone (GnRH), and dopamine (DA)^1^ (Fig. 1). CRH and TRH neurons within the PVH activate the HPA and HPT axes to stimulate the release of ACTH and thyroid-stimulating hormone (TSH), respectively, from the adenohypophysis. The release of ACTH results in the release of glucocorticoids from the adrenal gland, and the release of TSH induces secretion of thyroid hormones from the thyroid gland^1^. The HP-somatotropic (HPS) axis is bidirectionally controlled by hypothalamic neuroendocrine cells with opposite functions. This axis is upregulated by GHRH neurons in the ARH, which initiates the release of growth hormone (GH) from the adenohypophysis and insulin-like growth factor-1 (IGF-1) from the liver^24,25^. Conversely, SST neurons in the periventricular nucleus (PeV) and PVH downregulate the HPS axis by inhibiting GH secretion^25,26^. The HP-gonadal (HPG) axis is stimulated by GnRH neurons in the preoptic area (POA), which causes secretion of follicle-stimulating hormone (FSH)/luteinizing hormone (LH) and sex hormones^1^. DAergic neurons in the PeV and ARH downregulate prolactin (PRL) release^1,27^, whereas several neurohormones including TRH and GnRH trigger PRL release^28,29^. In contrast to DA, which is an amine, hypothalamic neurohormones are peptide hormones.

Hormones of the HP axes are associated with multiple metabolic functions. For example, estradiol is a gonadal steroid hormone released by stimulation of the HPG axis. Studies have shown that food intake is reduced close to the time of ovulation and that postmenopausal women gain body weight; therefore, estradiol was proposed to exert anti-obesity effects^30^. Later, investigators attributed the anti-obesity effects of estradiol to estrogen receptor-α expressed by pro-opiomelanocortin (POMC) neurons within the ARH and steroidogenic factor-1 neurons within the ventromedial nucleus of the hypothalamus (VMH)^31^. As mentioned earlier, hormones controlled by the HPA and HPT axes are known to regulate appetite and metabolism^4,5,8,9^. Compared with the functions of hormones released by the neurohypophysis and the other HP axes, the “classical” function of glucocorticoids and thyroid hormones is more relevant to the regulation of energy balance and glucose homeostasis. Interestingly, glucocorticoids and thyroid hormones promote food intake^4,5^, whereas CRH and TRH produce anorexigenic effects^8,9^. These findings suggest a complex nature of appetite and metabolism regulation by the HPA and HPT axes. Recent studies have observed that both CRH and TRH neurons within the PVH release glutamate to regulate homeostasis^13,14^, which highlights the non-neuroendocrine function of these neurohormone-expressing cells. Therefore, investigators believe that the “neuroendocrine” subsets of CRH and TRH neurons utilize the HPA and HPT axes, whereas other “non-neuroendocrine” cell populations affect appetite and metabolism via neural circuitry. In the following sections, we review the available literature to discuss the role of CRH neurons/the HPA axis and TRH neurons/the HPT axis in the regulation of appetite and metabolism, with a focus on the differences in the functions of each hormone and distinctions between neural and endocrine regulation.

## Role of the HPA axis and CRH neurons

CRH is a 41-amino acid peptide that is widely expressed throughout the body, including the brain^32,33^. Available evidence suggests that in addition to playing a pivotal role in stress-related responses^34^, CRH actively participates in the regulation of energy metabolism^35^. Most CRH neurons that control the HPA axis are located within the PVH, particularly in the lateral part^36^. CRH neurons within the PVH may affect appetite and metabolism either intrinsically or via HPA axis stimulation^37,38^. Additionally, CRH neurons located outside the hypothalamus may function independent of the HPA axis. We discuss the control of appetite and metabolism by the HPA axis and the CRH neurons expressed throughout the brain.

### The HPA axis

Glucocorticoids are secreted from the adrenal cortex following HPA axis activation. Corticosterone (CORT), which is the major glucocorticoid in rodents, induces hyperphagia and increases fat mass and body weight^39–42^ (Table 1). Chronic administration of exogenous CORT concomitant with high-fat diet (HFD) feeding did not cause further increases in fat mass and body weight^43,44^, although obesity secondary to HFD feeding is associated with elevated blood CRH, ACTH, and CORT levels. Exogenous CORT administration induced whitening of brown adipose tissue (BAT), which is associated with downregulation of uncoupling protein-1 (UCP-1) expression, as well as expansion of white adipose tissue (WAT)^42^. CORT administration was shown to elevate plasma triglyceride (TG) levels and increase the liver TG content^39,42^, which was associated with increased expression of genes involved in hepatic fatty acid metabolism. CORT injections also tended to increase the blood free fatty acid (FFA) level, although the difference was not statistically significant^42^. However, energy expenditure was unchanged in mice after CORT administration, regardless of whether the mice were fed a HFD or a chow diet^40,43^. Locomotive activity remained unchanged^43^ or was decreased^39^ after exogenous CORT administration. These results suggest that CORT-induced obesity is largely due to increased appetite rather than changes in energy consumption. Studies suggest that the orexigenic effects of glucocorticoids are at least partially mediated by modulation of POMC and AgRP neurons within the ARH^41,45^.

**Table 1:** Metabolic effects of exogenous HPA axis hormones.

Hormone	Application routes (animal model)	Food intake	Body weight/fat and lean mass	Energy expenditure parameters	Glucose balance	Lipid metabolism	Ref.
CORT	In drinking water (mouse)	↑	↑ Body weight ↑ Gonadal WAT mass	↓ Locomotive activity	↑ Insulin ↔ Glucose (after 2 weeks) ↑ Glucose (after 4 weeks)	↑ Blood TG	^39^
	In drinking water (mouse)	N.D.	↔ Body weight	↔ VO_2_ ↔ Locomotive activity	↔ GTT ↔ ITT ↔ Insulin	N.D.	^43^
	In drinking water (mouse)	↑	↑ Body weight ↑ Fat mass ↓ Lean mass	↔ RER	N.D.	N.D.	^40^
	Via pellet implantation (mouse)	↑	↓ Body weight gain ↑ BAT and WAT mass (↓ UCP-1)	N.D.	↔ Glucose	↑ Blood TG ↑ Liver TG ↔ Blood FFA (a non-significant increase)	^41,42^
ACTH	s.c. injection (rat)	↓	↓ Body weight	N.D.	↔ Glucose	↔ Blood TG ↔ Blood FFA ↔ Blood Total cholesterol ↔ Blood HDL cholesterol ↑ Blood LDL cholesterol	^47,51^
CRH	i.c.v. injection (rat)	↓	↓ Body weight ↓ Retroperitoneal and epididymal fat pads	↑ VO_2_ ↓ RER ↑ Locomotive activity ↑ Sympathetic activity	↔ Glucose	N.D.	^8,35,52,53^
	PVH injection (rat)	↓	N.D.	N.D.	N.D.	N.D.	^54^
	LS injection (rat)	↓	↔ Body weight	N.D.	N.D.	N.D.	^55^

*N.D*. not determined, see text for abbreviations.

Previous studies have also reported the effects of exogenous CORT on glucose metabolism. It is well known that glucocorticoid-induced gluconeogenesis and glycogenolysis result in increased blood glucose levels^38^. Dexamethasone (a glucocorticoid analog) has been reported to reduce insulin secretion from the isolated pancreas^46^. However, the effects of glucocorticoids on whole-body glucose homeostasis are equivocal. It was shown that CORT administration for 1 to 2 weeks did not affect glucose homeostasis^39,42^; however, CORT administration for 4 weeks caused hyperglycemia with increased insulin levels^39^, although glucose homeostasis was unaffected after 22 weeks of CORT treatment^43^. These findings suggest that although glucocorticoids may cause hyperglycemia, they appear to produce variable effects on long-term glucose homeostasis depending on the experimental conditions.

Interestingly, the effects of ACTH on appetite and energy consumption contrasted with those induced by glucocorticoids (Table 1). For example, subcutaneous ACTH injections administered for a month caused anorexia and reduced body weight^47^. In addition, treatment of primary cultures of BAT and WAT with ACTH led to elevated UCP-1 levels^48^. The effects of ACTH on lipid and glucose metabolism were also different from those produced by glucocorticoids. ACTH was shown to cause a transient increase in insulin secretion from the isolated pancreas^49^ and release of FFAs from isolated epididymal fat tissue^50^ under ex vivo conditions. However, ACTH injections administered in vivo for 3 consecutive days did not affect blood glucose, TG and FFA levels^51^. Blood levels of total cholesterol and high-density lipoprotein (HDL) cholesterol were also unchanged by in vivo ACTH injections, although this treatment increased plasma CORT and low-density lipoprotein (LDL) cholesterol levels^51^. Currently, no plausible explanation is available for the disparate observations; however, these results could be attributed to the possibility that exogenous ACTH affects the HPA axis and glucocorticoid secretion under some experimental conditions. Further studies utilizing knockout or knockdown of endogenous ACTH, as described below for CRH, are required to resolve this issue.

The effects of exogenous CRH on energy homeostasis are also markedly different from those of CORT, although these effects are consistent with those of exogenous ACTH administration (Table 1). For example, intracerebroventricular (i.c.v.) administration of CRH resulted in reduced appetite, decreased body weight gain, reduced fat pad weight, and increased sympathetic activity without any effects on blood glucose levels^8,52,53^. Another study demonstrated that i.c.v. injections of CRH increased oxygen consumption (VO_2_) and locomotion concomitant with a decrease in the respiratory exchange ratio (RER)^35^. CRH injections into the PVH reduced food intake in rats^54^, and CRH injections into the lateral septal area (LS) caused anorexia in food-deprived mice with no effects on body weight^55^. However, CRH-deficient mice did not show any defects in feeding behavior^56,57^ or even showed anorexigenic phenotypes^58,59^, which suggests that experiments using exogenous CRH may not accurately predict the metabolic effects of endogenous CRH. These results also emphasize the necessity of loss-of-function experiments to corroborate the findings of experiments using exogenous hormones to investigate the role of the HPA axis in the regulation of appetite and metabolism.

### CRH neurons

Studies in mice showed that chemogenetic inhibition or toxin-induced ablation of PVH CRH neurons did not alter acute or chronic food intake or body weight^60,61^ (Table 2). Interestingly, increased excitatory synaptic input onto PVH CRH neurons was associated with the anorexigenic effects of glucagon-like peptide-1 (GLP-1) signaling that originates in the nucleus tractus solitarius (NTS), and chemogenetic inhibition of PVH CRH neurons significantly attenuated the anorexigenic effects of GLP-1 signaling^62^. In this study, optogenetic stimulation of PVH CRH neurons, which presumably mimics the effects of GLP-1 signaling, suppressed food intake^62^. In another study, impaired activity of PVH CRH neurons exaggerated neuropeptide Y (NPY)-induced hyperphagia^63^. It was recently reported that fasting-induced activation of AMP-activated kinase (AMPK) causes increased activity of PVH CRH neurons, which contributes to a preference for a high-carbohydrate diet (nutrition) over a HFD (palatability) under fasting conditions^64^. A more recent study demonstrated that HFD feeding blunted the responsiveness of PVH CRH neurons and that mice with blunted PVH CRH neuronal responsiveness were more likely to develop HFD-induced obesity^65^. Overall, these studies suggest that PVH CRH neurons may participate in the fine tuning of food intake rather than control food intake per se. It is currently unknown whether appetite modulation by PVH CRH neurons occurs via the action of hormones or neurotransmitters. Considering the conflicting reports on the role of endogenous CRH in appetite regulation^56–59^, it can be inferred that the observed alterations in food intake are largely mediated by glutamate, which is a classical neurotransmitter released by PVH CRH neurons. Notably, defective glutamatergic neurotransmission from single-minded-1 neurons, which constitute the largest proportion of PVH neurons, was shown to cause obesity^66^. PVH CRH neurons send monosynaptic projections to various brain regions^13,67^ and polysynaptic projections to peripheral tissues and organs that regulate metabolism, such as WAT and the liver^68^. Therefore, defective glutamatergic neurotransmission from PVH CRH neurons is also likely to play a role in the pathophysiology of obesity.

**Table 2:** Metabolic phenotypes of CRH and TRH neuronal activity modulation.

Target nucleus	Neuronal population (animal model)	Modulation of neuronal activity (viral construct)	Involved neural circuitry	Observed phenotype	Ref.
PVH	CRH neuron (Crh-ires-cre mouse)	Chemogenetic inhibition (AAV8-DIO-hM4Di-mCherry)	N.D.	↔ Food intake	^60^
	CRH neuron (Crh-ires-cre mouse)	Neuronal ablation (AAV-DJ-CMV-DIO-eGFP-2A-TeNT)	N.D.	↔ Food intake ↔ Body weight	^61^
	CRH neuron (Crh-ires-cre mouse)	Chemogenetic activation (AAV-hSyn-DIO-hM3Dq-mCherry)	N.D.	↓ Food intake	^62^
		Chemogenetic inhibition (AAV-hSyn-DIO-hM4Di-mCherry)	N.D.	↓ Anorexia by GLP-1	
	TRH neuron (Trh-ires-cre mouse)	Chemogenetic activation (AAV8-DIO-hM3Dq-mCherry)	Glutamatergic innervation of AgRP neurons within the ARH	↑ Food intake	^14^
CeA	CRH neuron (Crh-ires-cre mouse)	Optogenetic activation (AAV2-EF1α-DIO-ChR2-EYFP)	N.D.	↔ Food intake	^73^
	CRH neuron (Crh-ires-cre mouse)	Chemogenetic activation (AAV2-hSyn-DIO-hM3Dq-mCherry)	N.D.	↔ Food intake (basal) ↓ Food intake (with stress)	^77^
BNST	CRH neuron (Crh-ires-cre mouse)	Chemogenetic activation (AAV5-EF1α-DIO-hM3Dq-mCherry)	N.D.	↔ Food intake ↔ Body weight	^78^

*N.D*. not determined, see text for abbreviations.

Outside the PVH, CRH is highly expressed by neurons of the central amygdala (CeA) and the bed nucleus of the stria terminalis (BNST)^69^. Conventionally, CRH neurons in the CeA and BNST have received considerable attention for their roles in the development of fear and anxiety^70,71^. Available evidence suggests that CRH neurons in these regions also participate in the control of appetite and metabolism. A recent study demonstrated that lentiviral knockdown of CRH in the CeA resulted in elevation of the basal but not the stress-induced CORT level^72^, which suggests that CeA CRH neurons downregulate the basal (non-stress) activity of the HPA axis. These neurons do not directly project to the median eminence; therefore, it is possible that CRH neurons in the CeA inhibit PVH CRH neurons via an unidentified neural circuitry. A previous study reported that photostimulation of CeA CRH neurons did not change food consumption under either fed or fasting conditions^73^ (Table 2). Moreover, administration of RO27-3225 (an MC4R agonist) and ghrelin failed to induce the expression of Fos, a marker for increased activity, in these neurons^74^. These results suggest that CeA CRH neurons do not control food intake. However, CRH expression was selectively decreased in rats, which developed compulsive eating upon introduction of palatable food after conditioning with prolonged intermittent presentation of a chow diet and palatable food^75^. In addition, fast-refeeding paradigms caused elevated CeA CRH neuronal activity in mice fed either a chow diet or a HFD^74,76^. A more recent study showed that chemogenetic activation of CeA CRH neurons exacerbated novelty-induced suppression of food consumption, whereas food intake and locomotion in the home cage remained unaffected^77^. Therefore, it can be concluded that CRH neurons in the CeA are dispensable for feeding behavior under normal conditions, although they respond to some stressful conditions to control food intake.

Chemogenetic activation of BNST CRH neurons did not change body weight or food intake^78^, similar to the findings associated with CeA CRH neurons (Table 2). However, optogenetic stimulation of BNST GABAergic neuronal projections resulted in feeding phenotypes. It was previously shown that optogenetic stimulation of BNST GABAergic neurons (Vgat^BNST^ neurons) to LHA projections induced food consumption in fed mice, whereas optogenetic inhibition of this circuit diminished food consumption in fasted mice^79^. Similarly, food consumption was increased by optogenetic stimulation of axon terminals of Vgat^BNST^ neurons innervating PBN neurons in sated mice^80^. Notably, this manipulation increased the consumption of salty and bitter-tasting food^80^. BNST CRH neurons are primarily GABAergic; however, it is unclear whether stimulation and inhibition of BNST CRH neuronal projections to the above-mentioned brain areas also affect feeding behavior under comparable experimental conditions.

## Role of the HPT axis and TRH neurons

Thyroid hormones released from the thyroid gland include thyroxine (T4), which is converted by the action of deiodinase enzymes into the bioactive form triiodothyronine (T3). Thyroid hormones affect the metabolic rate and synthesis of proteins essential for thermogenesis in cold environments predominantly via peripheral mechanisms^81^, but they may also affect feeding behavior via their actions on specific areas of the brain^82^. TRH neurons reportedly regulate appetite and metabolism directly via neural circuitry within the brain^14,83^. Currently, there is little direct evidence that TRH neurons control appetite and metabolism indirectly through the HPT axis. Below, we discuss the metabolic function of thyroid hormones, TSH, TRH, and TRH neurons in the brain.

### Thyroid hormones and TSH

Type 2 deiodinase (D2) is an important enzyme that catalyzes the conversion of endogenous T4 to T3. Local production of T3 in the hypothalamus is controlled by tanycytes, which are glial cells that express D2^84,85^. Fasting is reported to cause a significant increase in hypothalamic T3 and D2 mRNA levels/activity^86–88^. Conversely, D2-null mice showed reduced food intake in response to a fast-refeeding paradigm, but normal food intake was restored after exogenous T3 injections^89^. Therefore, increased production of hypothalamic T3 secondary to D2 activity is important for increased food intake in response to acute food deprivation.

Considering that body weight is usually reduced in hyperthyroidism^90^, increased food intake secondary to the action of thyroid hormones was previously considered a compensatory mechanism for the increased energy consumption in these patients. However, a direct action of T3 on hypothalamic neurons was suggested in a study in which exogenous T3 significantly increased food intake whether it was injected subcutaneously or directly into the VMH, whereas T3 injections into the ARH did not alter food intake^86^ (Table 3). However, subsequent studies demonstrated that food intake was unaffected by T3 injections into the VMH^91,92^. In another study, T3 was shown to activate UCP-2 and induce mitochondrial proliferation in orexigenic NPY/AgRP neurons within the ARH, which reportedly contributed to rebound feeding after food deprivation^89^. T3-induced stimulation of food intake was also attributed to enhanced hypothalamic AMPK activity^93^, although the specific neuronal population involved remains unclear. Therefore, current experimental evidence suggests that T3 increases food intake via actions in the medial basal region of the hypothalamus, including the VMH and ARH, but further studies are warranted to accurately identify the contributory neuronal populations and the relevant cellular mechanisms.

**Table 3:** Metabolic effects of exogenous HPT axis hormones.

Hormone	Application routes (animal model)	Food intake	Body weight	Energy expenditure parameters	Glucose balance	Lipid metabolism	Ref.
T3	s.c. injection (rat)	↑	↔	↔ VO_2_ (single or short-term injections) ↑ VO_2_ (long-term injections)	N.D.	N.D.	^86^
	i.c.v. injection (rat)	↔	↓	↑ BAT thermogenesis	N.D.	↔ Blood TG ↔ Blood FFA ↑ Liver TG	^91,92^
	VMH injection (rat)	↔ or ↑	↓	↑ BAT thermogenesis ↓ RER ↓ Locomotive activity	N.D.	↑ Blood TG ↑ Hepatic lipogenesis	^86,91,92^
TRH	s.c. injection (rat)	↓	↔	↑ Body temperature	N.D.	N.D.	^98^
	i.c. injection (rat)	↓	N.D.	N.D.	N.D.	N.D.	^99^
	i.c.v. injection (rat)	↓	N.D.	N.D.	↑ Glucose	N.D.	^9,99,100^
	i.c.v. injection (hamster)	N.D.	N.D.	↑ BAT temperature	N.D.	N.D.	^101^
	PVH injection (rat)	N.D.	N.D.	↑ Body temperature	↑ Glucose	N.D.	^102^
	VMH injection (rat)	↔	N.D.	↑ Locomotive activity	N.D.	N.D.	^104^
	VMH injection (hamster)	N.D.	N.D.	↑ BAT temperature	N.D.	N.D.	^101^
	DMH injection (hamster)	N.D.	N.D.	↑ BAT temperature	N.D.	N.D.	^101^
	LHA injection (rat)	↓	N.D.	↔ Locomotive activity	N.D.	N.D.	^104^
	LHA injection (hamster)	N.D.	N.D.	↔ BAT temperature	N.D.	N.D.	^101^

*N.D*. not determined, see text for abbreviations.

Thyroid hormones increase thermogenesis and energy consumption primarily via peripheral mechanisms^94^. However, i.c.v. injections as well as chronic subcutaneous injections of exogenous T3 were shown to increase energy expenditure in rats^86,91,92^ (Table 3), which suggests the involvement of central mechanisms. Furthermore, T3 injections into the VMH were shown to suppress AMPK function to increase sympathetic activity and promote BAT thermogenesis^91,92^. Notably, T3-induced suppression of AMPK signaling within the VMH increased vagal activity to increase hepatic lipogenesis and blood TG levels, whereas the decreased RER suggested that BAT utilized blood TGs for thermogenesis^92^. Overall, it appears that T3 suppresses AMPK signaling within the VMH, which at least partially contributes to T3-mediated promotion of thermogenesis via both the sympathetic and parasympathetic nervous systems.

Currently, limited data are available regarding the role of TSH in the regulation of metabolism. A recent clinical study reported that serum cholesterol and TG levels were positively correlated with TSH levels in children and adolescents with subclinical hypothyroidism, a condition characterized by elevated TSH but normal T4 levels^95,96^. This finding suggests that elevated TSH levels may be an early indicator of an adverse lipid profile. Another study showed that TSH receptor stimulation may promote WAT and BAT adipogenesis^97^. Therefore, it is reasonable to conclude that TSH may regulate fat metabolism and increase fat mass, although these effects appear to contradict the effects of thyroid hormones. Further studies are essential to conclusively establish the role of TSH.

### TRH and TRH neurons

A previous study showed that subcutaneous injections of exogenous TRH decreased food intake and increased body temperature in rats^98^ (Table 3). Additionally, intracranial (i.c.) and i.c.v. TRH injections suppressed food intake and increased BAT temperature and blood glucose levels^9,99–101^. TRH injections into the PVH caused a prompt increase in body temperature and blood glucose as well as CORT levels^102^. These studies suggest that TRH may inhibit food intake and reduce body weight, as well as stimulate metabolism, via central mechanisms. Exogenous TRH and T3 produced similar effects to increase energy expenditure; however, the anorexigenic effects of exogenous TRH were not consistent with the orexigenic effects of exogenous T3. Perhaps the anorexigenic effects of TRH are independent of the HPT axis; previous studies demonstrated that TRH-induced suppression of food intake was not accompanied by effects on TSH or thyroid hormone levels^9,103^. Interestingly, TRH injections into different areas of the hypothalamus yielded distinct phenotypes. For example, TRH injections into the VMH stimulated locomotion in rats; however, this effect was not observed after injections into the LHA^104^. TRH injections into the LHA reduced food intake; however, injections into the VMH did not affect food consumption^104^. A study performed in hamsters showed an increase in BAT temperature after TRH microinjections into the dorsomedial nucleus of the hypothalamus (DMH) or VMH but not into the LHA^101^. It is possible that exogenous TRH injected into a specific brain area can spread to adjacent brain sites; therefore, these findings should be confirmed using more sophisticated methods, such as studies in genetically engineered mice and neuron-specific viral injections.

Studies have investigated the cellular and circuit-level mechanisms underlying the development of in vivo phenotypes after exogenous TRH injections. Earlier research showed that TRH administration stimulated glucose-responsive neurons within the VMH^105,106^, which may explain the anorexigenic effects of i.c.v. TRH^9,99,100^. In another study, i.c.v. injections of TRH increased histamine turnover in the tuberomammillary nucleus, PVH, and VMH in rats, and the anorexigenic effects of i.c.v. TRH were attenuated in histamine-depleted rats and histamine H1 receptor-null mice^107^. TRH was also shown to suppress the activity of melanin-concentrating hormone (MCH) neurons in the LHA indirectly by increasing synaptic inhibition through activation of local GABAergic neurons^108^, which suggests that the metabolic effects of TRH can be attributed to inhibition of MCH neuronal activity. In this study, TRH was shown to excite the hypocretin/orexin neurons in the LHA, although it produced no effect on the activity of POMC or NPY neurons within the ARH^108^. Therefore, multiple cellular and synaptic mechanisms are implicated in the in vivo phenotypes resulting from exogenous TRH injections. Considering the TRH-induced alterations in the activity of multiple neuronal populations, future experiments should focus on assigning specific neuronal populations to distinct phenotypes of TRH administration.

TRH neurons are located in multiple hypothalamic areas, including the PVH, DMH, VMH, and LHA^109^. It was previously shown that chemogenetic activation of PVH TRH neurons stimulates feeding via their excitatory glutamatergic projections to the orexigenic AgRP neurons within the ARH^14^ (Table 2). Additionally, PVH TRH neurons project to the brain stem and spinal cord and activate UCP-1 in BAT to control thermogenesis and body temperature^83^. PVH TRH neurons are reported to function as targets of major metabolic signals, such as leptin and melanocortin^110^. In rat models, leptin was shown to regulate TRH production via a combination of a direct action on PVH TRH neurons and an indirect mechanism by which leptin first modulates the activity of ARH neurons, which consequently project to PVH TRH neurons^111^. Leptin was also reported to directly activate PVH TRH neurons and increase serum T4 levels in fasted mice^112^. However, a recent study reported that only a few PVH TRH neurons express leptin receptors and that PVH TRH neurons receive few axon fibers originating from ARH POMC neurons in mice^113^. It is, therefore, reasonable to infer that leptin-induced regulation of PVH TRH neurons shows species-specific differences. MC4R is expressed by most TRH neurons within the PVH^114^. PVH TRH neurons were shown to receive synaptic input from the POMC and NPY/AgRP neurons within the ARH, constituting the central melanocortin pathway^115–117^. In vivo studies have also confirmed that i.c.v. injections of α-melanocyte-stimulating hormone can prevent the reduction in *Trh* gene expression that occurs during fasting^114,118^. Therefore, the central melanocortin pathway originating from the ARH appears to control PVH TRH neurons via MC4Rs, although the physiological significance of MC4Rs expressed by TRH neurons remains to be determined.

Currently, little is known regarding the function of TRH neurons located outside the PVH. TRH neurons in the LHA were shown to receive input from the POMC and NPY/AgRP neurons within the ARH, which suggests that the activity of TRH neurons in the LHA is affected by the metabolic state of the organism^119^. However, no studies have investigated the metabolic functions of TRH neurons in the LHA. Further studies are warranted to accurately delineate the functions of TRH neurons located in the above-mentioned hypothalamic areas.

## Concluding remarks

Hypothalamic neurohormone-expressing cells control appetite and metabolism via hormones and neurotransmitters. Therefore, the mechanisms underlying their physiological functions are significantly more complicated than those underlying humoral or neural regulation. Various genetic tools are currently available to investigate the neural circuitry associated with various animal behaviors; these tools include genetically engineered mouse models and optogenetic, chemogenetic, and in vivo Ca^2+^ imaging techniques. In fact, these cutting-edge techniques have revealed several previously unknown mechanisms underlying homeostatic regulation by hypothalamic neurohormone-expressing cells at the molecular, cellular, and circuit levels. To date, these techniques have been applied primarily to understand the contribution of non-neuroendocrine or neural mechanisms to feeding behavior and metabolism. Currently available data regarding the contribution of endocrine mechanisms are derived mainly from experiments using more conventional methods, such as fluorogold labeling, immunostaining, and hormone measurements. Notably, the neuroendocrine effects of these cells are potentially more powerful and long-lasting than the non-neuroendocrine effects; therefore, it is necessary to address this issue using novel techniques with the understanding that these techniques could lead to major scientific breakthroughs. Therefore, further research is warranted to develop effective strategies to evaluate the endocrine and neural functions of hypothalamic neurohormone-expressing cells of interest to determine the relative importance of each aspect in the regulation of feeding behavior and metabolism. This information will enable scientists to gain a deeper understanding of the functions of neurohormone-expressing cells and facilitate the development of novel therapeutic strategies for obesity and metabolic diseases.
